# circSLC39A8 by sponging hsa-miR-11181-5p and direct binding to PIK3CA mRNA promotes retinoblastoma proliferation

**DOI:** 10.7150/jca.99599

**Published:** 2025-04-21

**Authors:** Jian Gong, Zicheng Song, Jiandong Pan, Xiangwei Huang, Xiaofen Feng, Qian Li

**Affiliations:** 1 Department of Clinical Laboratory, the Second Affiliated Hospital and Yuying Children's hospital of Wenzhou Medical University, Wenzhou, Zhejiang 325027, China.; 2 State Key Laboratory of Ophthalmology, Optometry and Vision Science, Eye Hospital, Wenzhou Medical University, Wenzhou, Zhejiang 325000, China.; 3 Pediatric Fundus Department, School of Optometry and Ophthalmology, Eye Hospital of Wenzhou Medical University, Wenzhou, Zhejiang 325000, China.; 4 The Key Laboratory of Pediatric Hematology and oncology Diseases of Wenzhou, The Second Affiliated Hospital & Yuying Children's Hospital of Wenzhou Medical University, Wenzhou, Zhejiang 325027, China.

**Keywords:** circRNA, miRNA, Dual regulation, PIK3CA, RB

## Abstract

**Background:** Retinoblastoma (RB) is a prevalent intraocular malignant tumor, posing a significant threat to human life and health. Although the involvement of circRNAs in various malignancies has been reported, their precise role in RB remains incompletely understood.

**Methods:** High-throughput sequencing was utilized to construct differential expression profiles of circRNAs, followed by candidate gene screening using RT-qPCR. The circular structure of circSLC39A8 was confirmed through stability assays with RNase R and Act D. A research system for transiently silencing and overexpressing circSLC39A8 was established, with flow cytometry employed to assess cell cycle and apoptosis levels. Bioinformatics analyses, RT-qPCR, and western blot experiments were conducted to evaluate the expression of circSLC39A8, hsa-miR-11181-5p, and PIK3CA. RNA antisense purification experiments were performed to elucidate interactions among circSLC39A8, hsa-miR-11181-5p, and PIK3CA.

**Results:** Our findings revealed a significant upregulation of circSLC39A8 in RB. Functionally, circSLC39A8 was identified as promoting cellular proliferation and suppressing apoptosis *in vitro*, thereby facilitating RB progression. Mechanistically, circSLC39A8 indirectly augmented the expression levels of PIK3CA mRNA by acting as a competitive endogenous RNA for hsa-miR-11181-5p, consequently enhancing the stability of PIK3CA mRNA and ultimately fostering RB cell proliferation while inhibiting apoptosis.

## Introduction

Retinoblastoma (RB) is the predominant intraocular malignancy affecting infants and children, characterized by malignant cell lesions in the anterior segment of ocular photoreceptors^1^,^2^. RB is prone to intracranial and distant metastases, posing a significant threat to affected children's lives. Despite the development of treatment modalities such as intravenous chemotherapy, intravitreal chemotherapy, radiotherapy, focal therapy, laser, and cryotherapy for retinoblastoma^3^,^4^, the overall survival rate remains unsatisfactory. Therefore, unraveling the molecular mechanisms underlying RB pathogenesis and exploring innovative diagnostic and therapeutic approaches holds paramount importance. While studies indicate that RB development involves genetics and epigenetics^5^, the precise molecular mechanism remains elusive.

Circular RNAs (circRNAs) are covalently closed circular RNA molecules formed through back-splicing, devoid of 5' caps and 3' tails^6^. Initially considered by-products of the splicing process and regarded as non-functional non-coding RNAs, ongoing research has revealed that circRNAs play pivotal roles in a diverse range of physiological and pathological processes within the human body. The unique circular structure of circRNAs confers resistance to RNA exonucleases, resulting in a longer half-life compared to linear RNAs. This exceptional stability positions circRNAs as distinctive biomarkers and promising therapeutic targets for various diseases^7^.

Accumulating evidence indicates the involvement of circRNAs in tumorigenesis and tumor progression, exerting their influence through diverse functional mechanisms that impact biological processes such as cell differentiation, proliferation, invasion, and apoptosis^8^. CircRNAs function as miRNA sponges, thereby modulating the post-transcriptional expression of target genes. For instance, circ-AKT3 can sequester miR-296-3p to inhibit E-cadherin expression and impede metastasis in clear cell renal cell carcinoma (ccRCC). Additionally, circRNAs possess the ability to directly interact with proteins, participating in subcellular localization and protein degradation. Notably, circZFR interacts with SSBP1 proteins, facilitating the assembly of CDK2/cell-cycle protein E1 complexes and thereby promoting cell-cycle progression and cell proliferation at an accelerated rate^9^. Additionally, circRNAs have the ability to directly bind to mRNAs, exerting influence on gene expression. A representative example is circZNF609 which interacts with CKAP5 mRNA, enhancing its stabilization and translation, consequently regulating microtubule dynamics and tumorigenicity^10^. Ongoing research has even revealed the potential of circRNAs in encoding small molecule peptides^11^.

Despite recent investigations into the impact of circRNAs on retinoblastoma development, the precise molecular mechanisms underlying circRNA-mediated regulation of RB development remain incompletely elucidated. Further exploration is warranted to ascertain whether circRNAs can effectively target tumor signaling pathways, thereby modulating RB development through diverse mechanisms.

The PI3K/AKT/mTOR pathway represents a highly conserved signal transduction network in all eukaryotic cells, crucial for regulating cell survival, growth, proliferation, and migration in response to external stimuli^12^,^13^. At the core of this pathway lie Class I phosphatidylinositol 3-kinase (PI3K) and protein kinase B (AKT) as pivotal functional proteins^14^. PI3K, an enzyme composed of dimers, phosphorylates phosphatidylinositol 4,5 diphosphate (PIP2) into phosphatidylinositol 3,4,5-trisphosphate (PIP3)^15^. PIP3 acts as a vital second messenger that activates downstream AKT signaling pathways and governing various biological processes such as cell proliferation, the cell cycle, and migration^16^,^17^.

Noteworthy findings suggest that the upregulation of miR-153 leads to the suppression of its target gene IGF1R, thereby inhibiting the activation of the Raf/MEK and PI3K/AKT signaling pathways, consequently impeding the proliferation and invasion of retinoblastoma cells^18^. The catalytic subunit of PI3K consists of four isoforms: p110α, p110β, p110γ, and p110δ; it also includes regulatory subunits^19^. The catalytic subunit α of phosphatidylinositol-4,5-bisphosphate 3-kinase (PI3KCA) encodes p110α, and its upregulation and mutation were initially identified in lung cancer^20^,^21^, a prevalent oncogene. Altered expression of PIK3CA leads to sustained activation of the PI3K/AKT signaling pathway independent of growth factors, thereby promoting tumor cell growth and invasion^22^. This findings suggest the potential involvement of genetic alterations in PIK3CA in retinoblastoma development.

Liu W et al. demonstrated that activation of circ-ZEB1, mediated by downregulation of miR-199a-3p, promotes the expression of PIK3CA, thereby influencing the proliferation and apoptosis of hepatocellular carcinoma^23^. Voutsadakis IA et al.'s investigation of PIK3CA mutations in colorectal cancer revealed their occurrence in 20-25% of colorectal cancers. Colorectal cancers with PIK3CA mutations exhibited higher total tumor mutation burdens (TMBs) compared to their unmutated counterparts, highlighting the frequent presence of PIK3CA gene mutations and suggesting potential for developing combination targeted therapies^24^. AKT, which is activated by PI3K or phosphatidylinositol-dependent kinase (PDK), exhibits dysregulated expression or activation in various cancers, including ovarian, lung, and pancreatic cancers, in the context of growth factors, inflammation, and DNA damage. Given its association with cancer cell proliferation and survival, AKT emerges as a pivotal target for cancer prevention and treatment^25^.

In our investigation, a significant up-regulation of circSLC39A8 expression was observed in retinoblastoma cells. Silencing circSLC39A8 yielded noteworthy outcomes, as it hindered the proliferative capacity and induced apoptosis in RB cells. Through bioinformatics analysis and detailed mechanistic studies, we discovered that circSLC39A8 functions as a molecular sponge for hsa-miR-11181-5p. This interaction indirectly augmented PIK3CA mRNA levels while simultaneously targeting PIK3CA mRNA. Consequently, this intricate process enhanced the stability of PIK3CA mRNA, initiating the activation of the PI3K/AKT signaling pathway and ultimately fostering the proliferation of RB cells. Our findings highlight the pivotal role of circSLC39A8 in modulating the delicate equilibrium between proliferation and apoptosis in RB cells. Furthermore, our study elucidates the dual regulatory mechanism wherein circRNAs function as molecular sponges, intricately interacting with downstream mRNAs, thereby driving the progression of RB. These insights offer novel perspectives for unraveling the intricate mechanisms governing RB development and pave the way for exploring innovative therapeutic strategies for this condition.

## Materials and Methods

### Cell culture

Human retinal epithelial cells (ARPE-19), human retinal glioma cells (WERI-Rb-1), human retinoblastoma cells (Y79), and 293T cells were procured from the Type Culture Collection (ATCC), Cellcook Biotech (Guangzhou, China), and the National Collection of Authenticated Cell Cultures (Shanghai, China). ARPE-19 cells were nurtured in DMEM/F-12 medium (Servicebio, G4610). WERI-Rb-1 cells were cultured in RPMI-1640 medium (Servicebio, G4534) supplemented with 10% fetal bovine serum (Gibco, 10099141C) and 1% penicillin-streptomycin (Servicebio, G4003). Y79 cells were maintained in RPMI-1640 medium (Servicebio, G4534) supplemented with 20% fetal bovine serum (Gibco, 10099141C) and 1% penicillin-streptomycin (Servicebio, G4003). 293T cells were cultured in DMEM medium (Servicebio, G4511) supplemented with 10% fetal bovine serum (Gibco, 10099141C) and 1% penicillin-streptomycin (Servicebio, G4003). All cells were incubated at 37°C in a 5% CO_2_ incubator.

### RNA isolation and quantitative real-time PCR (qPCR) assays

RNA extraction from tissues or cells was performed using TRIzol^®^ Reagent (Invitrogen, 15596018). The GoScript^TM^ Reverse Transcription System Kit (Promega, A5002) was used for reverse transcription as per the provided instructions. For quantitative polymerase chain reaction (qPCR), the GoTaq® qPCR Master Mix Kit (Promega, A6002) was utilized following the specified guidelines. GAPDH served as an internal reference, and relative expression levels were calculated using the 2^-ΔΔCt^ method. Primer synthesis was conducted by Sangon Biotech (Shanghai, China), and the primer sequences are detailed in Supplementary Table S1.

### Plasmids

The circSLC39A8 overexpression plasmid was meticulously designed by the author, while the PIK3CA overexpression plasmid and their corresponding control empty plasmid were constructed by Fenghui Biotechnology (Hunan, China). Plasmid sequences were verified through sequencing analysis. The transformed DH5α cells were cultured in lysogeny broth (LB) medium at 37°C for 16 hours. Subsequently, the plasmid were extracted using the Plasmid Midi Kit (QIAGEN, 12145), following the manufacturer's instructions.

### Cell transfection

The riboFECT CP Transfection Kit (166T) (RiboBio, C10511-05) was used the transfection of siRNA into cells according to the manufacturer's instructions. All siRNA and scramble sequences were synthesized by Guangzhou RiboBio (China), and their sequences are listed in Table S1. For overexpression plasmid transfection, the Lipofectamine 3000 Transfection Kit (Invitrogen, L3000015) was used following the recommended protocol.

### Construction of stable transgenic cell lines

The lentivirus-mediated construction and packaging of the stable silencing circSLC39A8 (circSLC39A8 sh) plasmid vector was performed. Y79 cells were infected with the packaged lentivirus, followed by a 48-hour incubation period, and subsequently, the fluorescence signal was visualized using a fluorescence microscope (AMG EVOS, Mill Creek, WA, USA). The establishment of stable cell lines was meticulously achieved through monoclonal screening method, and the efficiency of overexpression was assessed via qPCR analysis.

### RNase R treatment

For RNase R treatment, 1 μg of total RNA was incubated at 37°C for 10 minutes, with or without the addition of 3 U/μg of RNase R. Subsequent procedures included reverse transcription and qPCR analysis using circSLC39A8 and SLC39A8 primers.

### Stability testing

The Y79 cells were seeded in 6-well plates at a density of 450,000 cells per well. After 18 hours of culture, the cells were treated with 2 μg/mL of Actinomycin D (Act D) (Sigma, SBR00013, Epicenter Biotechnologies, USA). The TRIzol cell suspension was collected at 0 hours, 4 hours, 8 hours, and 12 hours. RNA extraction followed by reverse transcription and qPCR analysis was performed to determine the expression levels of circSLC39A8 and SLC39A8.

### Cell nucleocytoplasmic separation experiment

We prepared 1×10^7^ Y79 cells and WERI-Rb-1 cells, respectively, and performed nuclear and cytoplasmic separation experiments using the PARIS™ Kit (Invitrogen, AM1921). The subcellular localization of circSLC39A8 was determined by employing qPCR technique. GAPDH was utilized as a cytoplasmic marker, while U6 served as a nuclear marker.

### Flow cytometric analysis of cell apoptosis

The Annexin V-FITC/PI Apoptosis Detection Kit (KeyGen Biotech, KGA107) was employed to quantify cell apoptosis. Following collection and a single wash with PBS, the cells were resuspended in Binding buffer. Subsequently, 5 μL of Annexin V-FITC and 5 μL of PI were sequentially added. The mixture was then incubated in the dark for 15 minutes and 5 minutes, respectively. Flow cytometry using CytoFLEX (Beckman Coulter) was utilized to determine the apoptosis rate for each experimental group.

### Flow cytometric cell cycle analysis

The Cell Cycle Detection Kit (KeyGen Biotech, KGA511) was employed in accordance with the manufacturer's instructions to analyze the cell cycle using a flow cytometer. Initially, transfected cells were collected, centrifuged, resuspended, fixed, and then subjected to another round of centrifugation followed by washing. Subsequently, they were incubated with 30 μL of RNase A for 30 minutes and stained with PI for 30 minutes in the absence of light. The cell cycle distribution of each experimental group was determined using a CytoFLEX flow cytometer (Beckman Coulter).

### Western blotting and antibodies

Cell lysate (10 mM Tris-HCl, pH 7.4, 1% SDS, 1 mM Na_3_VO_4_) was used to lyse cells, collect cellular proteins, and perform ultrasonic crushing. Protein concentration was measured using the Pierce^TM^ BCA Protein Assay Kit (Thermo Fisher Scientific, 23227). A 6-10% dodecyl sulfate-polyacrylamide gel was used to separate an equal amount of protein, and the protein was subsequently transferred to a polyvinylidene fluoride membrane (PVDF). After blocking non-specific binding with 5% skim milk, the primary antibody was incubated overnight at 4°C. Subsequently, the secondary antibody was incubated at 4°C for 3 hours. Chemiluminescence imaging was carried out using the Integrated Chemiluminescence Imager (Clinx, ChemiScope S6, Shanghai, China), and the gray value of the protein band was analyzed using Image J software. The antibody information used in this study is as follows: PI3KCA (Proteintech, 60318-1-Ig), AKT1 (Santa Cruz Biotechnology, sc-5298), p-AKT (S473) (Cell Signaling Technology, 4060T), β-actin (Affinity, AF7018), and β-tubulin (Servicebio, GB11017-100). Secondary antibodies include goat anti-mouse IgG (Proteintech, SA00001-1) and anti-rabbit IgG (Cell Signaling Technology, 7074S).

### mRNA stability test

Y79 cells were cultured in 6-well plates at a density of 4×10^5^ cells per well. Upon reaching approximately 90% confluency, Actinomycin D (Act D) (Sigma, SBR00013-1 mL, USA) was added at a concentration of 2 μg/mL. Cell RNA was collected at time points of 0 h, 4 h, and 8 h. Subsequently, TRIzol reagent (Invitrogen) was added to each well for RNA extraction, and the degradation rate of mRNA was quantified using quantitative polymerase chain reaction (qPCR).

### RNA antisense purification (RAP)

The oligonucleotides complementary to the target RNA were biotinylated using the RNA Antisense Purification (RAP) Kit (BersinBio, Bes5103-3) for capturing the relevant RNA. Initially, circSLC39A8 and PI3KCA RAP probe groups were designed with their sequences provided in Supplementary Table S1. A total of 1×10^8^ cells were collected and cross-linked at room temperature using a vertical mixer. Subsequently, the cells were lysed after collection, followed by addition of the RAP probe group post-DNA removal. Hybridization was performed at 37°C for 30 minutes. Each RAP sample probe was then added to prepared streptavidin-labeled magnetic beads and incubated on a vertical mixer at room temperature for 30 minutes. Finally, elution and purification of RNA were carried out, followed by amplification of target genes using qPCR. Agarose gel electrophoresis was conducted to analyze the data.

### Statistical analysis

The statistical analysis was performed using SPSS 25.0 (IBM, Chicago, USA), while data visualization and mapping were conducted using GraphPad Prism 8.3 (San Diego, USA). The mean ± standard deviation was used to present the study's data. For normally distributed measurement data, a *t*-test or analysis of variance (for three groups or more) was employed. Non-normally distributed measurement data were analyzed using the rank sum test. Count data between groups were compared using the chi-square test. Bivariate correlation of variables with a normal distribution was assessed through Pearson correlation analysis, whereas Spearman correlation analysis was utilized for variables with non-normal distributions. All tests were two-sided and *P* < 0.05 were considered statistically significant.

## Results

### Circular RNA circSLC39A8 screening and circular structure identification

**Figure 1:**
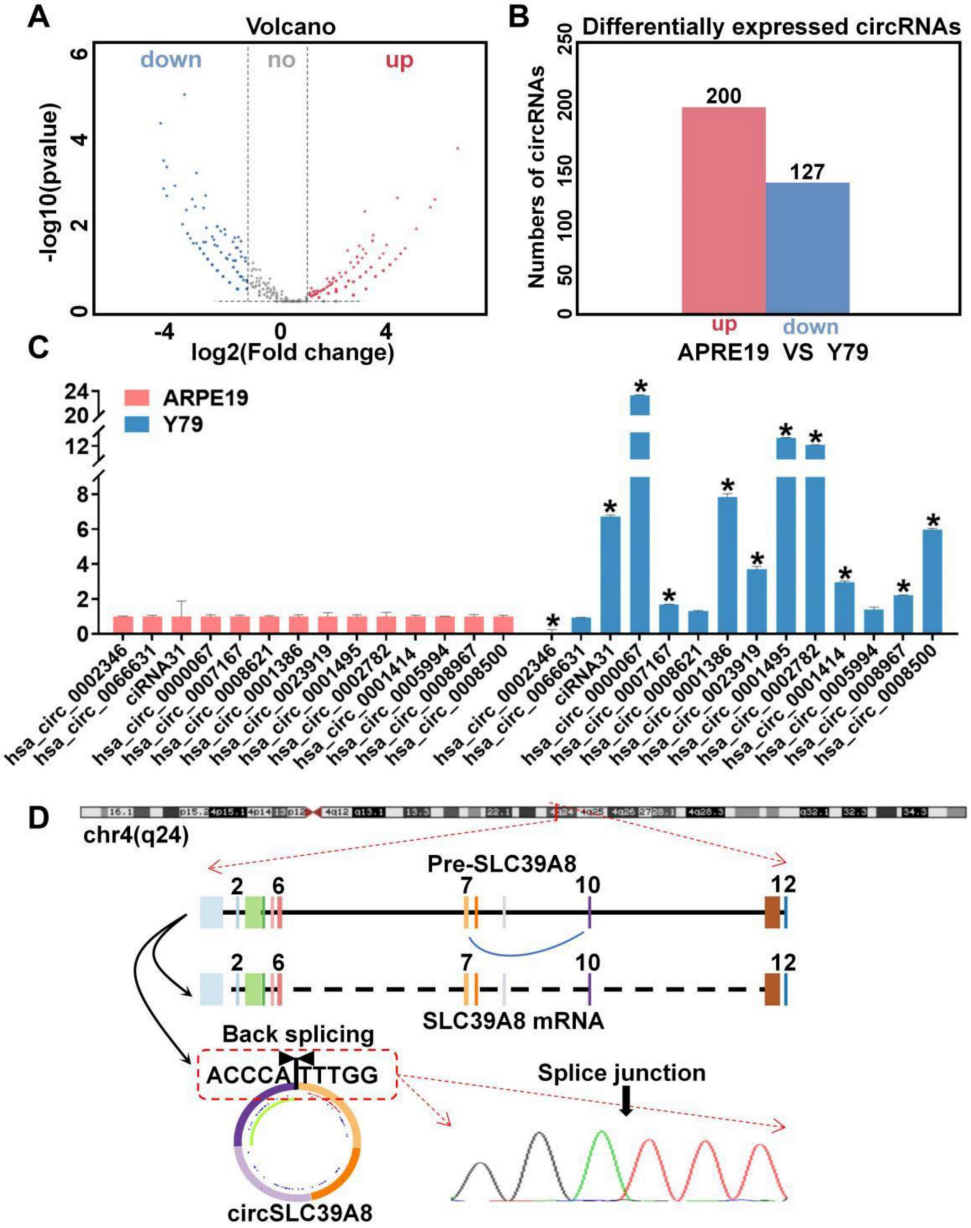
** Expression and characterization of circSLC39A8 in RB. (A)** Volcano plot illustrating the circRNA sequencing results in RB cells compared to human retinal epithelial cells. **(B)** Display of differentially expressed circRNAs from circRNA sequencing. **(C)** Quantitative PCR (qPCR) assessment of the 14 circRNAs with the most significant differential expression. **(D)** Schematic representation depicting the generation of circSLC39A8, accompanied by Sanger sequencing results of PCR products. * *P* < 0.05.

High-throughput sequencing was performed using human retinal epithelial cells (ARPE-19) as the control group and retinoblastoma cells (Y79) as the experimental group (Figure S1 A). Circular RNA (circRNA) differential expression profiles were established, revealing a total of 327 differentially expressed circRNAs (127 down-regulated circRNAs and 200 up-regulated circRNAs) (Figure 1 A-B).

Gene Ontology (GO) enrichment analysis of the differentially expressed circRNAs identified 14 significantly up-regulated circRNAs in the sequencing results. Subsequently, we examined the expression levels of these circRNAs in ARPE-19 and Y79 cells, which revealed a significant up-regulation of circSLC39A8 (hsa_circ_0002782) specifically in Y79 cells (Figure 1 C, Figure S1 B).

The gene structure analysis conducted on the UCSC website (https://genome.ucsc.edu/) revealed that circSLC39A8 originated from the reverse shearing of exons 7 and 10 within the SLC39A8 precursor gene. The circularization site of circSLC39A8 was further confirmed through Sanger sequencing of qPCR amplification products (Figure 1 D).

### *In vitro* proliferative capacity of circSLC39A8 in RB cells

**Figure 2:**
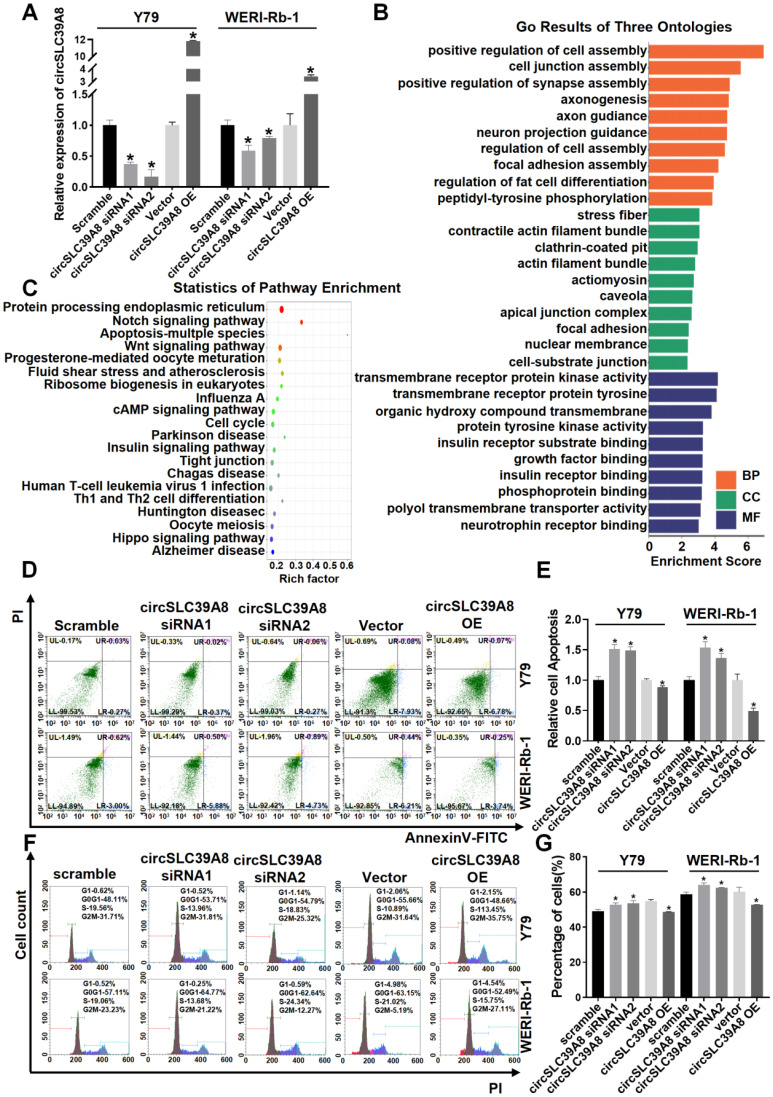
** CircSLC39A8 inhibits malignant proliferation of RB cells *in vitro*. (A)** Quantification of transient silencing and overexpression efficiency of circSLC39A8 through qPCR. **(B)** Gene Ontology (GO) analysis of differentially expressed circRNAs from circRNA sequencing. **(C)** Pathway enrichment analysis of differentially expressed circRNAs from circRNA sequencing. **(D)** Flow cytometry assessment of apoptosis following transient silencing and overexpression of circSLC39A8. **(E)** Statistical analysis of apoptosis detection results. **(F)** Flow cytometry analysis of cell cycle progression after transient silencing and overexpression of circSLC39A8. **(G)** Statistical summary of apoptosis detection results. * *P* < 0.05.

To elucidate the biological function of circSLC39A8 in RB, we designed small interfering RNA (siRNA) (Supplementary Table S1) and constructed a transient overexpression plasmid for circSLC39A8. The efficiency of silencing and overexpression of circSLC39A8 was assessed after transient transfection. The results demonstrated significant silencing of circSLC39A8 with the specific siRNA, while the overexpression plasmid significantly increased its expression (Figure 2 A). These findings confirm the successful establishment of a transient research system for investigating circSLC39A8's silencing and overexpression.

KEGG and GO analyses of differential expressed circRNAs from circRNA sequencing results revealed that circSLC39A8 is involved in molecular functions (MF) such as transmembrane receptor protein kinase activity and transmembrane receptor protein tyrosine, participating in biological processes (BP) including positive regulation of cell assembly and cell junction assembly. It is also associated with cellular components (CC) such as stress fiber and contractile actin filament bundles (Figure 2 B, Figure S2). Pathway enrichment statistics indicated the potential involvement of circSLC39A8 in several crucial tumor signaling pathways, including cAMP, cell cycle, apoptosis, etc. (Figure 2 C, Figure S3).

Subsequently, we conducted flow cytometry analysis to investigate the impact of transient silencing and overexpression of circSLC39A8 on apoptosis and cell cycle regulation. The results demonstrated that transient silencing of circSLC39A8 significantly induced apoptosis in Y79 and WERI-Rb-1 cells, whereas transient overexpression effectively suppressed apoptosis in these cells (Figure 2 D-E). Moreover, transient silencing of circSLC39A8 promoted G0/G1 phase progression in Y79 and WERI-Rb-1 cells, while overexpression of circSLC39A8 inhibited G0/G1 phase progression in these cells (Figure 2 F-G). Collectively, our findings from these cellular experiments provide evidence supporting the role of circSLC39A8 in enhancing RB cell proliferation ability while inhibiting apoptosis.

### circSLC39A8 down-regulates PIK3CA expression via the hsa-miR-11181-5p sponge

**Figure 3:**
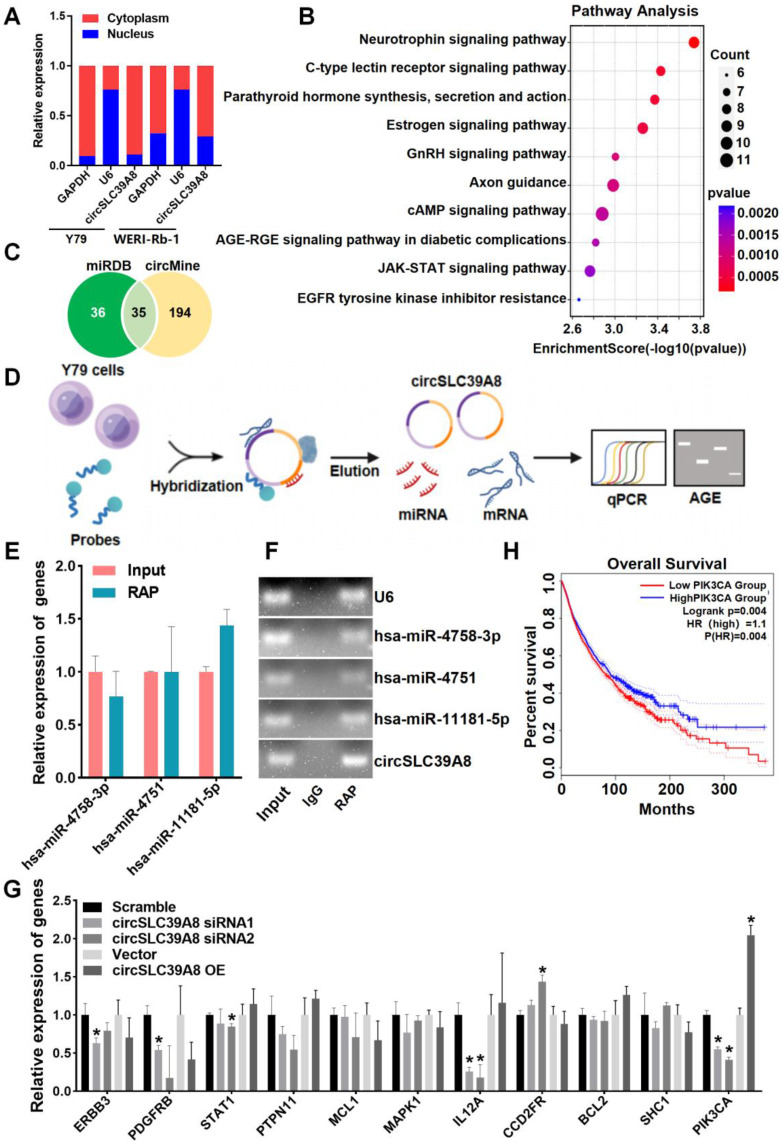
** CircSLC39A8 down-regulates PIK3CA expression via the hsa-miR-11181-5p sponge. (A)** Subcellular localization analysis of circSLC39A8 through nuclear and cytoplasmic experiments. GAPDH and U6 served as cytoplasmic and nuclear positive controls, respectively. **(B)** Enrichment analysis of pathways associated with circSLC39A8 downstream genes. **(C)** Prediction of miRNAs downstream of circSLC39A8 through miRDB, circMine website, and intersection analysis. **(D)** Schematic representation of RNA antisense purification (RAP) experiments. **(E)** qPCR detection of miRNA enrichment after RAP experiments conducted in Y79 cells. **(F)** Agarose gel electrophoresis results of RAP-qPCR products.** (G)** qPCR assessment of downstream gene expression levels after transient silencing and overexpression of circSLC39A8. **(H)** Overall Survival analysis. * *P* < 0.05.

The subcellular localization of circRNA is intricately linked to its molecular mechanism of action. Therefore, we performed subcellular localization analysis of circSLC39A8 in Y79 and WERI-Rb-1 cells. Nucleoplasmic fractionation experiments revealed the presence of circSLC39A8 in both the nucleoplasm and cytoplasm, with a predominant localization in the cytoplasm (Figure 3 A). To elucidate the underlying molecular mechanism by which circSLC39A8 promotes RB proliferation, we identified its interactions with various pathways, including cAMP, EGFR, and GnHR, through KEGG and GO enrichment analysis of downstream genes associated with circSLC39A8 (Figure 3 B).

Further bioinformatics analysis, utilizing miRDB and circMine database predictions, identified 35 miRNAs that potentially interact with circSLC39A8 (Figure 3 C). Among these, hsa-miR-4758-3p, hsa-miR-4751, and hsa-miR-11181-5p exhibited the most robust binding ability. RAP-qPCR experiments in Y79 cells confirmed the interaction between circSLC39A8 to all three miRNAs, with particularly significant binding observed for hsa-miR-11181-5p (Figure 3 D-F).

Subsequently, bioinformatics tools (Targetscan, miRDB, miRbase) were employed to predict potential target genes of hsa-miR-11181-5p. Among them, 11 candidate genes with the highest binding affinity were selected for further investigation. Silencing circSLC39A8 in Y79 cells resulted in a significant reduction in PIK3CA expression as determined by qPCR analysis (Figure 3 G). These findings suggested that circSLC39A8 functions as a competitive endogenous RNA for hsa-miR-11181-5p and consequently enhances PIK3CA expression. To elucidate the potential role of PIK3CA in RB and its correlation with patient prognosis, overall survival analysis was performed using data from RB patients obtained from the TCGA database. The results revealed that low PIK3CA expression was associated with prolonged overall survival and higher survival rate, whereas high PIK3CA expression correlated with shorter overall survival and lower survival rate. The difference between the two groups was statistically significant (Figure 3H).

### circSLC39A binds directly to PIK3CA mRNA and promotes its stability

**Figure 4:**
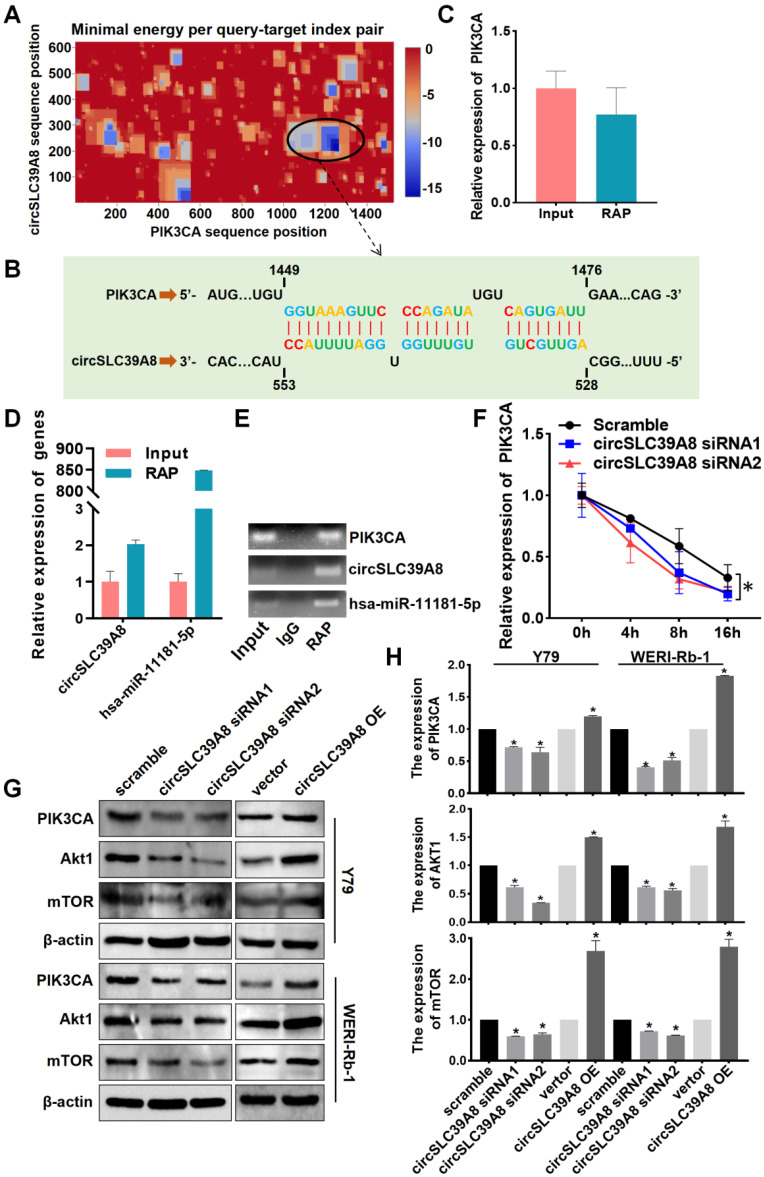
** CircSLC39A8 binds directly to PIK3CA mRNA and enhances its stability. (A)** Analysis of circSLC39A8 and PIK3CA mRNA binding clustering using the IntaRNA website.** (B)** Schematic illustration of circSLC39A8 and PIK3CA binding sites. **(C)** qPCR detection of PIK3CA expression in the RNA antisense purification (RAP) assay. **(D)** qPCR analysis of circSLC39A and hsa-miR-11181-5p expression levels in RAP experiments.** (E)** Agarose gel electrophoresis results of qPCR products from RAP experiments. **(F)** PIK3CA mRNA stability after transient silencing of circSLC39A8 assessed by qPCR following Actinomycin D (ActD) treatment in Y79 cells. **(G, H)** Western blot detection of PIK3CA and Akt protein expression levels post transient silencing and overexpression of circSLC39A8 in Y79 and WERI-Rb-1 cells. * *P* < 0.05.

The regulation of downstream mRNAs by circRNAs as miRNA sponges constitutes a pivotal mechanism underlying their biological functions. Moreover, circRNAs have been demonstrated to directly target mRNAs, thereby influencing mRNA stability. To investigate the potential targeting of PIK3CA mRNA by circSLC39A, we employed IntaRNA-RNA-RNA interaction analysis software and identified robust binding between circSLC39A and PIK3CA mRNA at multiple base binding sites (Figure 4 A-B). RAP experiments utilizing a PIK3CA-specific RAP probe set further validated the interaction between circSLC39A and PIK3CA mRNA, along with successful pull-down of circSLC39A and hsa-miR-11181-5p (Figure 4 C-E). These findings collectively indicate an intricate interplay among PIK3CA, circSLC39A, and hsa-miR-11181-5p.

To investigate the impact of circSLC39A directly binding to PIK3CA mRNA on PIK3CA expression, we conducted mRNA stability experiments. The results demonstrate that circSLC39A enhances the stability of PIK3CA mRNA, leading to an upregulation in the expression level of PIK3CA (Figure 4 F). Furthermore, transient silencing and overexpression of circSLC39A in Y79 and WERI-Rb-1 cells revealed a positive regulatory effect of circSLC39A on the expression of PI3KCA and AKT, as observed through Western blot experiments (Figure 4 G-H). Collectively, these findings indicate that circSLC39A binds directly to PIK3CA mRNA, promotes its stability, and activates the PI3K/AKT pathway.

### circSLC39A promotes RB proliferation and inhibits apoptosis through PIK3CA

**Figure 5:**
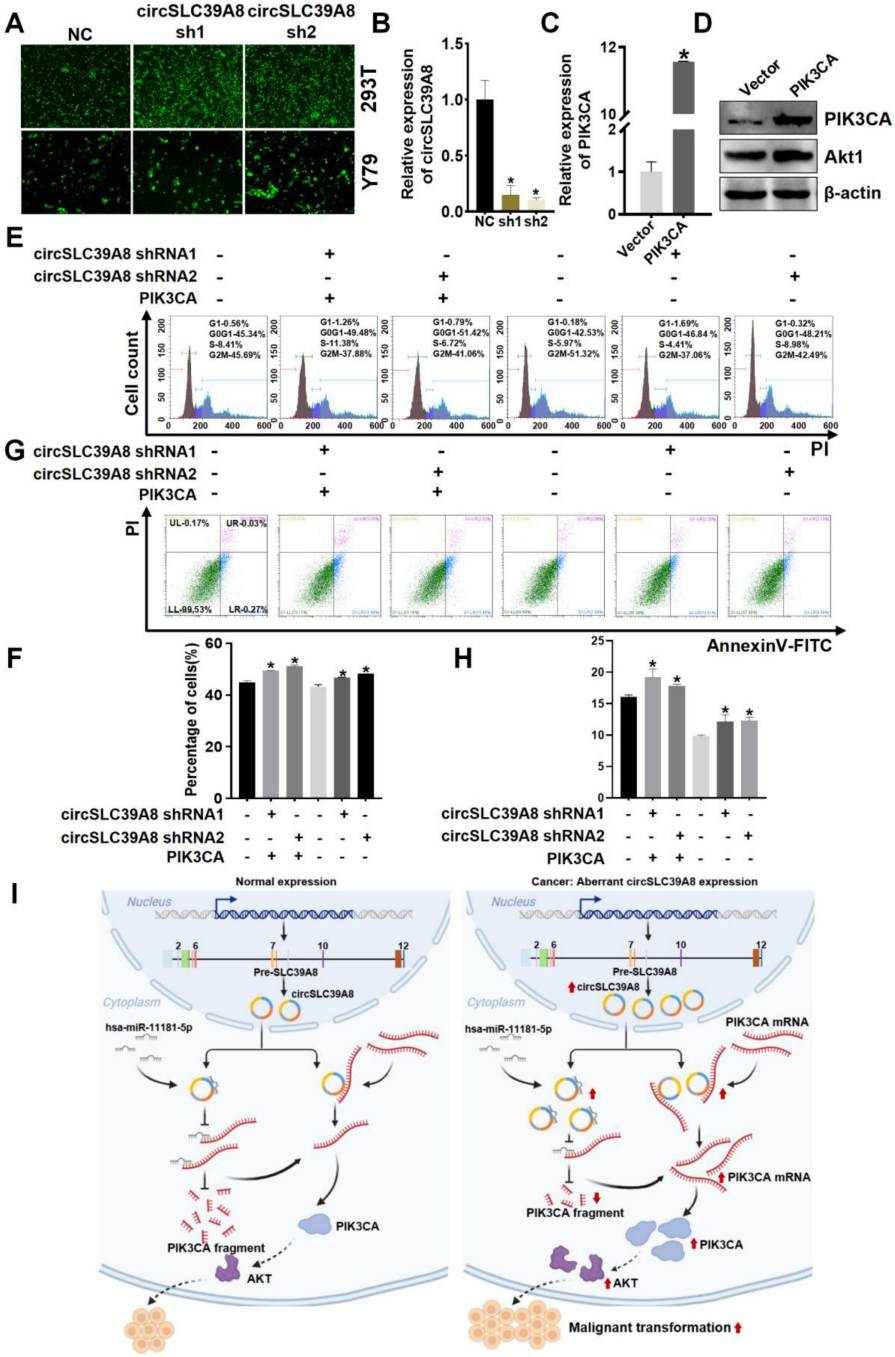
** CircSLC39A8 inhibits RB progression through the PI3K/AKT axis. (A)**Transfection of a circSLC39A8 stable silencing plasmid vector into 293T and Y79 cells followed by fluorescence detection. **(B)** qPCR assessment of circSLC39A8 stable silencing efficiency. **(C)** qPCR detection of PIK3CA plasmid overexpression efficiency. **(D)** WB analysis of PIK3CA and Akt protein expression levels after PIK3CA overexpression.** (E)** Flow cytometry detection of cell cycle after circSLC39A8 stable silencing and PIK3CA overexpression.** (F)** Statistical analysis of cell cycle detection results. **(G)** Flow cytometry assessment of cell apoptosis following circSLC39A8 stable silencing and PIK3CA overexpression. **(H)** Statistical analysis of apoptosis results. **(I)** Mechanism diagram. * *P* < 0.05.

To further investigate the role of circSLC39A in promoting RB progression through regulation of PIK3CA stability, we generated a stable Y79 cell line with silenced circSLC39A8 and established an overexpression system for PIK3CA using a PIK3CA overexpression plasmid (Figure 5 A-D, Figure S4). Transfection of Y79 cells with the PIK3CA overexpression significantly upregulated AKT (Figure 5 D).

Subsequent cell rescue experiments involved the overexpressing of PIK3CA while stably silencing circSLC39A8. Flow cytometry analysis revealed that the inhibitory effect on cell proliferation caused by circSLC39A8 silencing could be reversed by PIK3CA overexpression (Figure 5 E-H). These findings suggest that circSLC39A8 functions as a competitive endogenous RNA for hsa-miR-11181-5p, thereby enhancing PIK3CA expression. Furthermore, circSLC39A8 directly interacts with PIK3CA, promoting PIK3CA mRNA stability and exerting dual regulatory effects to promote RB cell proliferation while inhibiting apoptosis.

## Discussion

RB is a highly aggressive intraocular malignancy that predominantly affects children under the age of 3, often manifesting with a familial genetic predisposition. It can occur unilaterally or bilaterally, representing the most prevalent intraocular malignancy in infants and young children^26^. The global incidence of RB is around 9,000 new cases annually, with approximately 1,100 cases in China^27^,^28^. Although RB incidence exhibits no significant variations by geography, gender, race or eye type, regional disparities in economic conditions and medical resources contribute to substantial discrepancies in survival rates and ocular preservation. While some developed countries achieve nearly 100% survival rates, China reports a survival rate of approximately 84%^29^,^30^. Therefore, early diagnosis and standardized treatment are crucial for enhancing RB outcomes.

In recent years, the role of circular RNAs (circRNAs) in regulating various malignant tumors has garnered significant attention^31^. However, limited studies have delved into the biological functions and mechanisms of circRNAs in RB, resulting in a partial understanding of their involvement. This study identified circSLC39A8 as a markedly up-regulated circRNA in RB. The investigation unveiled that circSLC39A8 operates through a novel dual regulatory mechanism by enhancing PIK3CA mRNA stability, activating the PI3K/AKT pathway, and ultimately promoting RB development.

circRNAs, characterized by their circular structure, represent a distinct class of non-coding RNAs. Despite being discovered earlier, they initially received limited attention due to technological constraints in the previous century. However, with the advancement in high-throughput sequencing technology, circRNAs have regained prominence as a subject of research interest. They exhibit widespread expression across organisms and play diverse roles in various physiological and pathological processes^32^. Unlike linear RNAs, circRNAs primarily arise through alternative splicing events involving precursor RNAs encoding protein genes. Their closed-loop structure, formed by direct connections between the 5' and 3' ends, confers enhanced stability as they are less susceptible to degradation mediated by nucleic acid endonucleases.

Currently, two primary approaches are utilized for the detection of circular structure in circRNAs: Sanger sequencing and RNA linear digestion (RNase R) processing. In Sanger sequencing, specific qPCR primers are designed to target the reverse shear site of circRNA, ensuring that the primer spans across the shear site. The amplified qPCR product is subsequently subjected to sequencing analysis to validate its integrity^33^. On the other hand, RNase R digestion method plays a crucial role in confirming the circular nature of circRNAs due to their resistance towards RNA linear digestion enzymes. This technique involves treating circRNAs with RNA linear digestion enzymes followed by qPCR-based detection, which demonstrates the enhanced stability exhibited by circRNAs compared to linear RNAs^34^. In this study, we constructed a differential expression profile of circRNAs between RB cells and human retinal epithelial cells using high-throughput sequencing. Among the differentially expressed circRNAs, circSLC39A8, identified as a novel circRNA, exhibited significant up-regulation. To validate the expression of circSLC39A8 in RB cell lines, we performed experiments including qPCR with specific primers for circSLC39A8 and Sanger sequencing. These findings provide confirmation of the circular structure of circSLC39A8 and lay the foundation for subsequent investigations.

Numerous studies have extensively elucidated the involvement of circRNAs in diverse biological processes such as cell proliferation and apoptosis, and their dysregulated expression plays a pivotal role in the pathogenesis of diverse diseases, including diabetes mellitus, various neoplasms, and cardiovascular diseases^35^,^31^. Furthermore, circRNAs have been implicated in the development of several malignancies like lung cancer, hepatocellular carcinoma, gastric cancer, and bladder cancer^36^-^39^. The intricate association between circRNA expression levels and tumorigenesis/progression enables them to function as either oncogenes or tumor suppressors^40^, thereby holding significant promise for tumor diagnosis and therapy^41^.

In the context of RB, previous studies have identified differentially expressed circRNAs that play crucial roles in key biological processes, including migration, invasion, proliferation, and apoptosis of RB cells, thereby significantly impacting disease progression^42^. Noteworthy examples include circ_0000034, which accelerates RB development by upregulating ADAM19 expression, and circDHDDS, significantly up-regulated in RBs, promoting retinoblastoma proliferation^43^,^44^. Additionally, Circ-E2F3, acts as a ceRNA for miR-204-5p has been found to enhance RB proliferation and metastasis while inhibiting RB apoptosis by regulating the expression of ROCK1^45^,^46^. These findings underscore the vital role of circRNAs in RB development. This study contributes to the expanding knowledge of circRNAs in RB by identifying circSLC39A8, which is significantly upregulated in RB cells, as a crucial factor that inhibits RB apoptosis and promotes cell proliferation. The observed biological functions of circSLC39A8 in promoting RB proliferation and inhibiting apoptosis at the *in vitro* level suggest its potential role as an oncogenic factor in RB development.

RB, a highly aggressive intraocular malignancy, poses significant health risks, yet its mechanism of development remains incompletely understood. While circRNAs have been acknowledged for their diverse mechanisms of action, most studies have predominantly focused on circRNAs regulating RB progression through single mechanisms, leaving the multifaceted regulatory mechanisms contributing to RB progression unexplored. This study unveils a novel molecular mechanism in which circSLC39A8 functions as a ceRNA for hsa-miR-11181-5p to regulate PIK3CA mRNA. Additionally, circSLC39A8 directly interacts with PIK3CA mRNA, thereby enhancing mRNA stability and promoting PIK3CA expression. This dual regulatory mechanism sheds light on the intricate role of circSLC39A8 in driving RB progression.

Current understanding of circRNA function primarily revolves around their involvement in ceRNA networks. In this study, bioinformatics analysis combined with RAP mechanism experiments revealed that circSLC39A8 significantly enriched hsa-miR-11181-5p, subsequently promoting PIK3CA expression. The role of the circRNA/hsa-miR-11181-5p/PIK3CA axis in cancer, particularly RB, remains unreported. Furthermore, circRNAs have been demonstrated to directly bind to mRNAs, exerting an influence on mRNA stability and subsequent protein expression. In this study, a combination of bioinformatics analysis, RAP experiments, and stability assays provided evidence that circSLC39A8 specifically interacts with PIK3CA mRNA, thereby enhancing its stability. In addition to their roles as miRNA sponges and mRNA stabilizers, circRNAs can engage in direct interactions with proteins, playing pivotal roles in tumorigenesis. DICER is necessary for the production of siRNA and mature miRNA precursors by processing long dsRNA. In this study, while we demonstrated a direct binding association between circSLC39A8 and PIK3CA through RAP experiments, we did not investigate the regulation of PIK3CA mRNA by circSLC39A8 following the knockout of the DICER required for miRNA maturation. Therefore, in future studies, we aim to further substantiate the conclusion that circSLC39A8 influences mRNA stability by directly binding to PIK3CA mRNA through more sophisticated experimental designs. For instance, circNOL10 interacts with SCML1, inhibiting its protein ubiquitination and affecting mitochondrial function, thereby influencing lung cancer progression^47^. By directly binding to STAT3 and facilitating its protein ubiquitination, circFOXP1 promotes apoptosis^48^. Future investigations will explore whether circSLC39A8 can not only regulate PIK3CA mRNA levels but also affect PIK3CA protein levels, providing a more comprehensive understanding of its role in promoting RB progression.

The PI3K/AKT pathway has emerged as a crucial player in the development of various cancers, supported by a plethora of studies^49^. For instance, Xiang et al. highlighted the regulatory role of the MicroRNA/PI3K/AKT axis in osteosarcoma-associated genes, influencing cancer progression^50^. Bertacchini et al. demonstrated that targeting the PI3K/Akt/mTOR pathway could induce pro-apoptotic and anti-proliferative effects on hematological malignancies^51^. In relation to RB, studies have emphasized the significance of the PI3K/AKT pathway. Down-regulation of microRNA-182 was found to inhibit RB by suppressing the PI3K/AKT pathway, while microRNA-153-3p was shown to impede RB cell growth and invasion through targeting the IGF1R/Raf/MEK and IGF1R/PI3K/AKT signaling pathways^52^,^18^. The pivotal role of PIK3CA in the PI3K pathway underscores its oncogenic potential in tumor development and its association with the prognosis of various malignancies^53^. Up-regulation or activating mutations in the PIK3CA gene result in AKT activation, promoting cell survival, proliferation, and growth signaling, while inhibiting apoptosis through the phosphorylation of key targets such as Bcl2, FKHR, TWIST1, CREB, and YB1^54^. In this study, we further elucidated the impact of circSLC39A8 on RB by uncovering its connection to the PIK3CA/AKT pathway. The observed reversal of inhibited cell proliferation and increased apoptosis upon PIK3CA overexpression following circSLC39A8 silencing, as confirmed by Western blot and salvage assays, provides mechanistic insights. This reversal underscores the role of circSLC39A8 in enhancing the stability of PIK3CA mRNA through a dual regulatory mechanism. By unraveling these intricate interactions, the study offers a comprehensive understanding of how circSLC39A8 contributes to RB development. This newfound knowledge holds potential implications for targeted therapeutic interventions in RB cases associated with dysregulated circSLC39A8 and aberrant PIK3CA/AKT pathway activity.

## Conclusions

In conclusion, this study has identified circSLC39A8, a previously unrecognized circular RNA that exhibits significant upregulation in RB cells. The investigation clearly demonstrates the crucial role of circSLC39A8 in promoting RB progression. Furthermore, the study elucidates a dual regulatory mechanism employed by circSLC39A8: it acts as a sponge for hsa-miR-11181-5p, indirectly upregulating PIK3CA mRNA; simultaneously, it interacts with PIK3CA mRNA to enhance its stability and increase its expression (Figure 5I). These findings provide novel insights into the intricate molecular mechanisms underlying RB development and offer valuable perspectives for identifying potential therapeutic targets. This study paves the way for further research aimed at deepening our understanding of the molecular intricacies of RB and developing innovative therapeutic strategies.

## Supplementary Material

Supplementary figures and tables.
