# Supplementary material for: circSLC39A8 by sponging hsa-miR-11181-5p and direct binding to PIK3CA mRNA promotes retinoblastoma proliferation
Source: J Cancer. 2025 Apr 21;16(8):2434–48. doi: 10.7150/jca.99599 (PMC12170501; doi:10.7150/jca.99599)
Supplement: Supplementary file 1 — Supplementary figures and tables. [file jcav16p2434s1.pdf]

Supplementary Information

Jian Gong[1], Zicheng Song[2], Jiandong Pan[3], Xiangwei Huang[1], Xiaofen Feng [3,#],Qian Li[1,4, #]

[#]These authors contributed equally to this work

[1]Department of laboratory Medicine, the Second Affliated Hospital and Yuying Children's hospital of Wenzhou Medical University, Wenzhou, Zhejiang 325027, China

[2]State Key Laboratory of Ophthalmology, Optometry and Vision Science, Eye Hospital, Wenzhou Medical University,Wenzhou, Zhejiang 325000, China

[3]Pediatric Fundus Department, School of Optometry and Ophthalmology , Eye Hospital of Wenzhou Medical University, Wenzhou, Zhejiang 325000, China

[4]The Key Laboratory of Pediatric Hematology and oncology Diseases of Wenzhou, The Second Affiliated Hospital & Yuying Children's Hospital of Wenzhou Medical University, Wenzhou, Zhejiang 325027, China

Correspondence:

Xiaofen Feng

Pediatric Fundus Department,School of Optometry and Ophthalmology, Eye Hospital of Wenzhou Medical University, Wenzhou, Zhejiang, 325027, China

Phone: + 86 13868889266

Email: fengxiaofen1983@163.com

Qian Li

Department of Laboratory Medicine

The Key Laboratory of Pediatric Hematology and Oncology Disease of Wenzhou,

The Second Affiliated Hospital & Yuying Children's Hospital of Wenzhou Medical University, Wenzhou, Zhejiang, 325027, China

Phone: + 86 13634242347

Email: lisunqian1@163.com

Table S1. Nucleic acid sequence information of the siRNA and PCR primer

siRNA	Sequence	
	sense	antisense
circSLC39A8 siRNA1	GUGGUAUCUCUACAGUGUUUATT	UAAACACUGUAGAGAUACCACTT
circSLC39A8 siRNA2	GUAUCUCUACAGUGUUUAACUTT	AGUUAAACACUGUAGAGAUACTT
circSLC39A8 siRNA3	CUACAGUGUUUAACUGCUGAATT	UUCAGCAGUUAAACACUGUAGTT

PCR primer	Sequence	
	Forward	Reverse
circSLC39A8	ATGCTATGCAAATCCTGCTGTCA	CACATGGTGCACTGAAACCG
SLC39A8	CCAGCGCATCGGTCCTATG	CTTCCTGCACCAAGTAAAGAAGT
GAPDH	ATCAATGGAAATCCCATCACCA	GACTCCACGACGTACTCAGCG
hsa_circ_0002346	GTCCCCCGGACAGCTATGAA	CTCAAAGGGATTGCTGCAGGT
hsa_circ_0066631	CCATGTTTCTGGACGCGGATT	TTCCACTCTCAGGGCCTAGT
ciRNA31	GGGGAAGCAAGTCCCTGTTTT	CGACAGTTACACCTACGCAGC
hsa_circ_0000067	TGTGGAACACCAAGCACCAC	CAGCTGCTTTTACTCAAAGGCATA
hsa_circ_0007167	CCTCCAAGATTCTGGTCTGCTC	GGAGCCATGGTCTGCGATTT
hsa_circ_0008621	ATAGCAACTGAGGGCTTCGTG	AGCAGCGGTCTAATGCACTG
hsa_circ_0001386	CCTTGGGCATCATCGGACTT	AGCCTCATCCCACGTCCTTA
hsa_circ_0005260	CCCATGTGAAAGAGGTCAGCC	GAGGCTGTCAGGGAATCGGA
hsa_circ_0023919	AGCTGCCCAATGATCTGCTTG	GCTAGGGGCCTCCCCATGTA
hsa_circ_0001495	GGAGAGGTTGATGTCGAGCAA	GGCACACAATTATTCCATTCACCA
hsa_circ_0001414	GCAGAATGGGGTGATCGCT	ACTGGAGCTGCTGGTGTAAA
hsa_circ_0005994	ACTACAACTCGGGGACCTGC	CACGTGAAACGAAGAACCCCA
hsa_circ_0008967	AGAGATCTCATACTTCCTCGCGT	TGATCTCTCATGTCCCCCACT
hsa_circ_0008500	CGACCTGAAGCTTCTTCTCAGC	AGAAACTGGGGAGTATCTGGCT
PIK3CA	CCACGACCATCATCAGGTGAA	CCTCACGGAGGCATTCTAAAGT
SHC1	TACTTGGTTCGGTACATGGGT	CTGAGTCCGGGTGTTGAAGTC
MAPK1	TACACCAACCTCTCGTACATCG	CATGTCTGAAGCGCAGTAAGATT

CCND2	ACCTTCCGCAGTGCTCCTA	CCCAGCCAAGAAACGGTCC
MCL1	TGCTTCGGAAACTGGACATCA	TAGCCACAAAGGCACCAAAAG
PTPN11	GAACTGTGCAGATCCTACCTCT	TCTGGCTCTCTCGTACAAGAAA
IL12A	CCTTGCACTTCTGAAGAGATTGA	ACAGGGCCATCATAAAAGAGGT
STAT1	CAGCTTGACTCAAAATTCCTGGA	TGAAGATTACGCTTGCTTTTCCT
PDGFRB	AGCACCTTCGTTCTGACCTG	TATTCTCCCGTGTCTAGCCCA
ERBB3	GGTGATGGGGAACCTTGAGAT	CTGTCACTTCTCGAATCCACTG

**RAP Probe**	**Sequence**
782-RAP-1	TGAGGACCAAAGTTATCATTTCCAAAGTGGGTATGACCATTCTGACCATAT
782-RAP-2	TATCATTTCCAAAGTGGGTATGACCATTCTGACCATATGTCTTTAATAACATC
782-RAP-3	GAGTTTTTTCTTGAGGACCAAAGTTATCATTTCCAAAGTGGGTATGACCATT
PIK3CA-RAP-1	GAATTGAGGTCCCTAAGATCCACAGCTTCTTTACAAACGTTCAGAATATTTCT
PIK3CA-RAP-2	TTATCTAATTTATTATATATGTGCTTTGGCAATTCTGGTGAAGATTCTACATT
PIK3CA-RAP-3	TACTAGCTGAATTAAATACTGAGAAAGTTTGTCATCTGTTAAATATTTTTCCA

**shRNA primer**	**Sequence**

shRNA1	**Forward:**<br>GATCCGGTGGTATCTCTACAGTGTTTACTCGAGTAAACACTG<br>TAGAGATACCACTTTTTTGGAAG<br>**Reverse:**<br>AATTCTTCCAAAAAAGTGGTATCTCTACAGTGTTTACTCGAGT<br>AAACACTGTAGAGATACCACCG
shRNA2	**Forward:**<br>GATCCGGTATCTCTACAGTGTTTAACTCTCGAGAGTTAAACA<br>CTGTAGAGATACTTTTTTGGAAG<br>**Reverse:**<br>AATTCTTCCAAAAAAGTATCTCTACAGTGTTTAACTCTCGAG<br>AGTTAAACACTGTAGAGATACCG

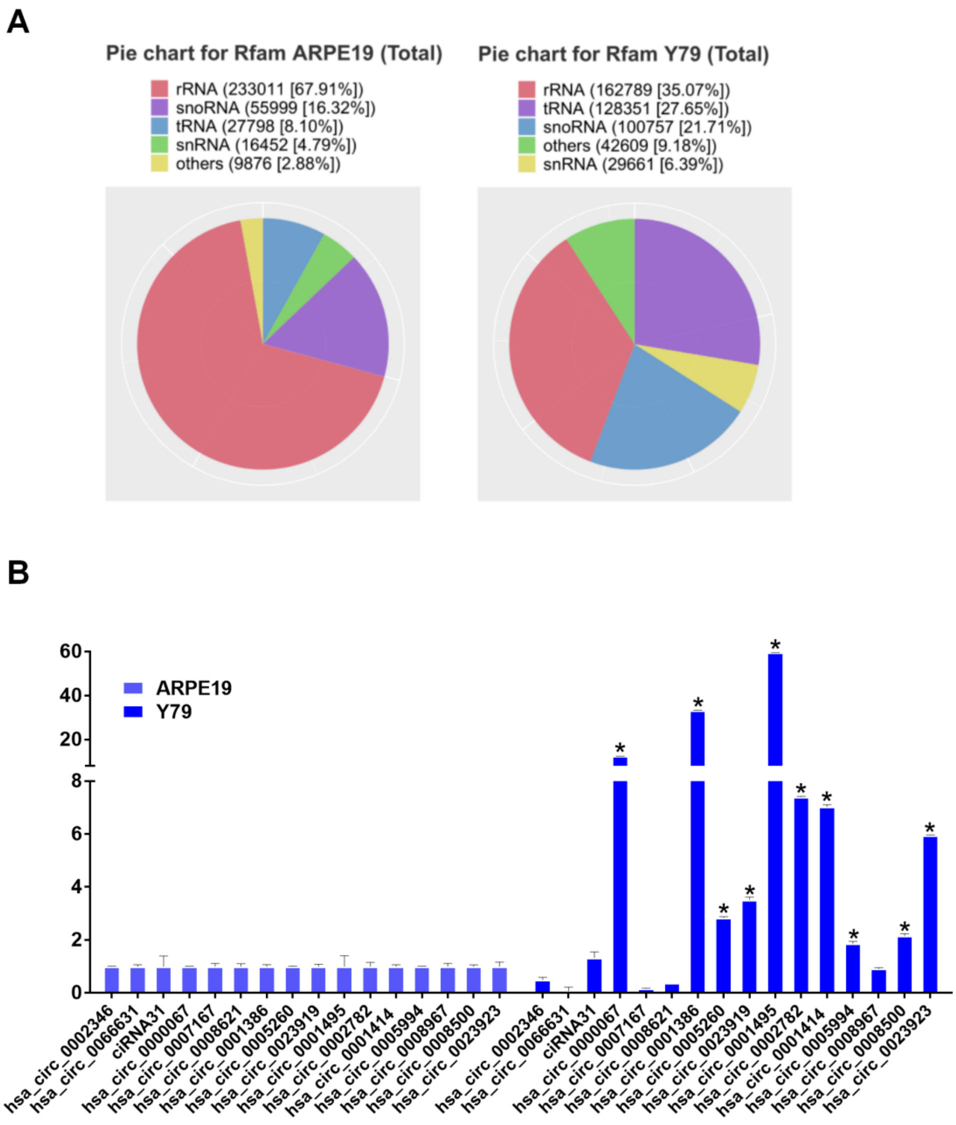

FigureS1 (A) Retinoblastoma cells were sequenced with human retinal epithelial cells for total

RNA classification. (B) qPCR detection of the most significantly differentially expressed 14 circRNA expression levels in duplicate experiments. * p<0.05.

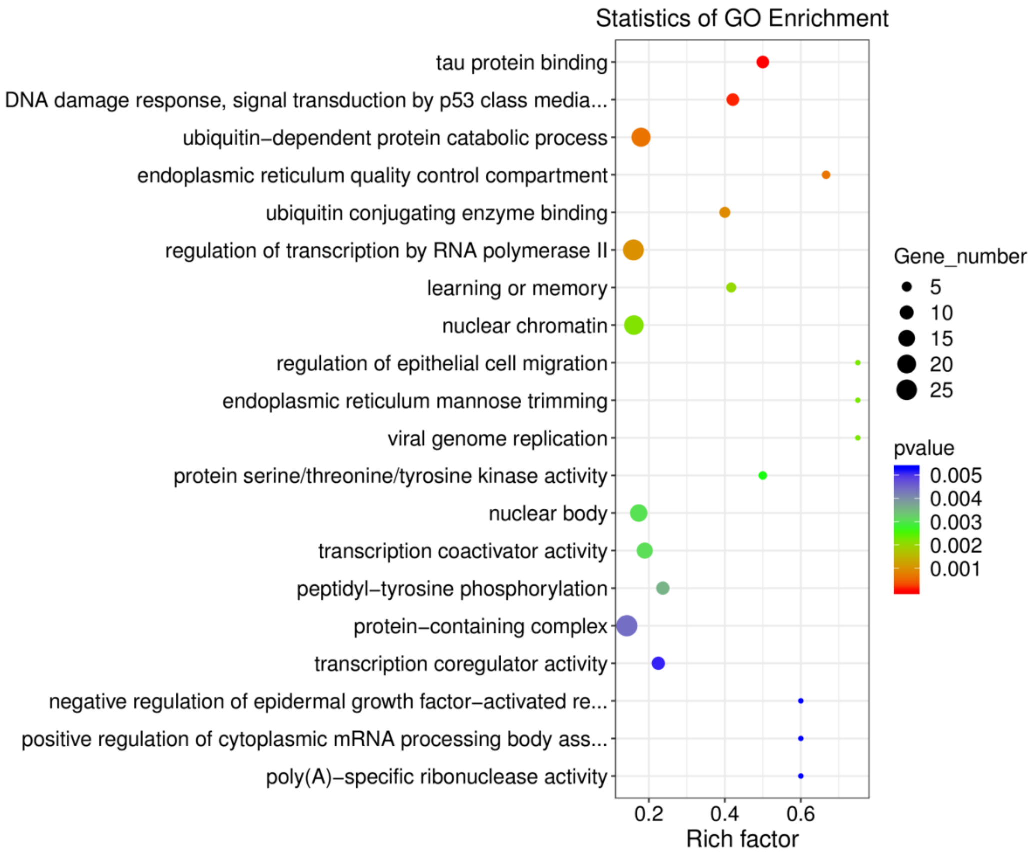

FigureS2 Go analysis of circSLC39A8 downstream genes.

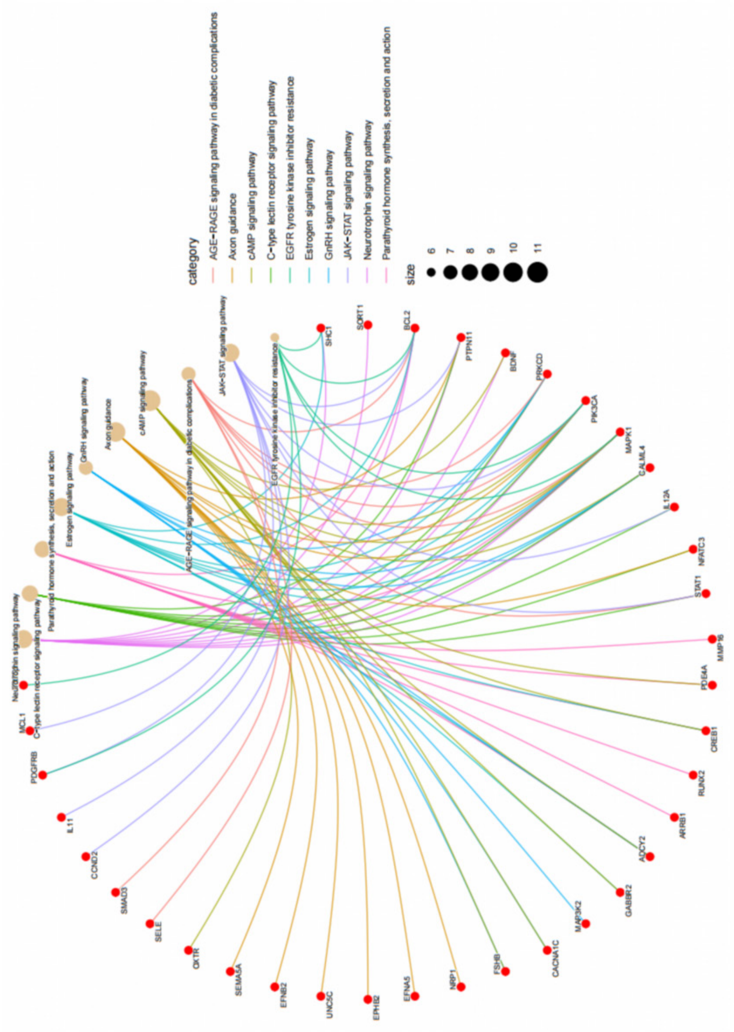

FigureS3 Pathway analysis of circSLC39A8 downstream genes.

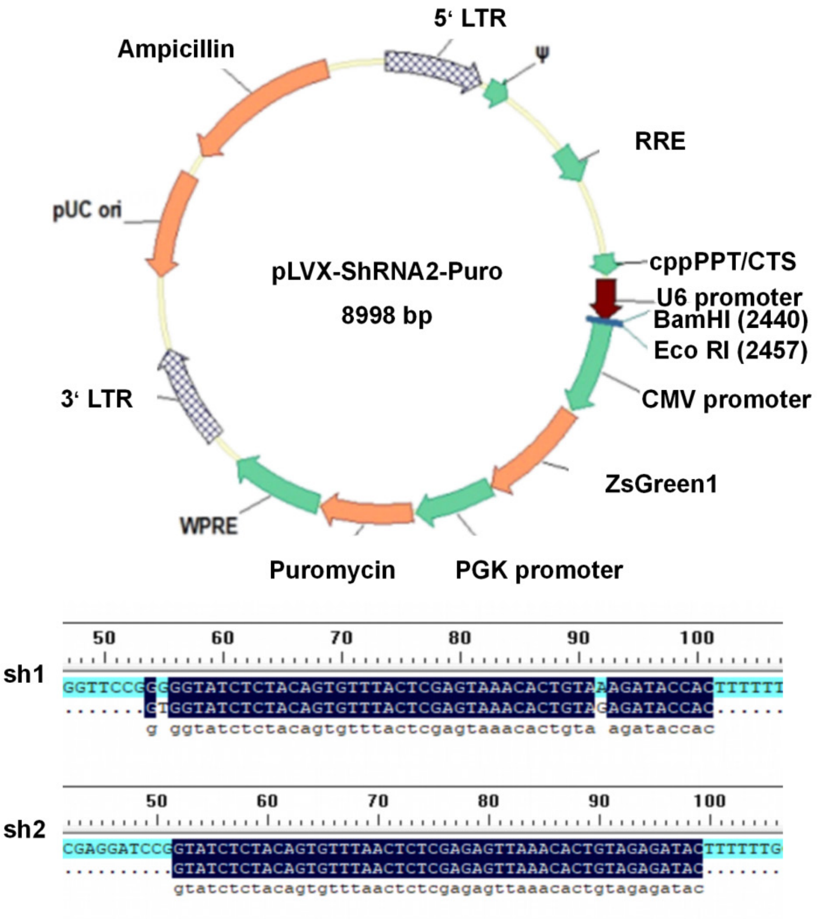

FigureS4 circSLC39A8 stable silencing plasmid vector mapping and construction of successful sequencing matching results.
